# Probiotic and Muscadine Grape Extract Interventions Shift the Gut Microbiome and Improve Metabolic Parameters in Female C57BL/6 Mice

**DOI:** 10.3390/cells12222599

**Published:** 2023-11-10

**Authors:** Tiffany M. Newman, Adam S. Wilson, Kenysha Y. J. Clear, E. Ann Tallant, Patricia E. Gallagher, Katherine L. Cook

**Affiliations:** 1 Department of Cancer Biology, Wake Forest University School of Medicine, Winston-Salem, NC 27157, USA; tiffanymnewman@gmail.com; 2 Department of Surgery-Hypertension, Wake Forest University School of Medicine, Winston-Salem, NC 27157, USA; awilson@wakehealth.edu (A.S.W.); kclear@wakehealth.edu (K.Y.J.C.); atallant@wakehealth.edu (E.A.T.); pgallagh@wakehealth.edu (P.E.G.); 3 Department of Physiology and Pharmacology, Wake Forest University School of Medicine, Winston-Salem, NC 27157, USA; 4 Comprehensive Cancer Center, Wake Forest University School of Medicine, Winston-Salem, NC 27157, USA

**Keywords:** muscadine grape extract, *Lactobacillus*, inflammation, macrophages, diet, fibrosis, gut microbiome, obesity, visceral adipose tissue

## Abstract

Obesity and Western-like diet consumption leads to gut microbiome dysbiosis, which is associated with the development of cardio-metabolic diseases and poor health outcomes. The objective of this study was to reduce Western diet-mediated gut microbial dysbiosis, metabolic dysfunction, and systemic inflammation through the administration of a novel combined intervention strategy (oral probiotic bacteria supplements and muscadine grape extract (MGE)). To do so, adult female C57BL/6 mice were fed a low-fat control or Western-style diet and sub-grouped into diet alone, probiotic intervention, antibiotic treatments, MGE supplementation, a combination of MGE and probiotics, or MGE and antibiotics for 13 weeks. Mouse body weight, visceral adipose tissue (VAT), liver, and mammary glands (MG) were weighed at the end of the study. Fecal 16S rRNA sequencing was performed to determine gut bacterial microbiome populations. Collagen, macrophage, and monocyte chemoattractant protein-1 (MCP-1) in the VAT and MG tissue were examined by immunohistochemistry. Adipocyte diameter was measured in VAT. Immunohistochemistry of intestinal segments was used to examine villi length, muscularis thickness, and goblet cell numbers. We show that dietary interventions in Western diet-fed mice modulated % body weight gain, visceral adiposity, MG weight, gut microbial populations, and inflammation. Intervention strategies in both diets effectively reduced VAT and MG fibrosis, VAT and MG macrophages, adipocyte diameter, and VAT and MG MCP-1. Interventions also improved intestinal health parameters. In conclusion, dietary intervention with MGE and probiotics modulates several microbial, inflammatory, and metabolic factors reducing poor health outcomes associated with Western diet intake.

## 1. Introduction

A calorically dense Western Diet (WD) contributed to the doubling of the obesity prevalence between 1980 and 2014 [1]. WD is rich in refined sugar, purified animal fats, processed meats, salt, and food additives and low in vitamins, minerals, and fiber [2]. When combined with a sedentary lifestyle, WD consumption is associated with chronic diseases including obesity-associated metabolic syndrome, type II diabetes mellitis, cardiovascular disease, non-alcoholic fatty liver disease, and cancer [2]. WD consumption has also been associated with chronic inflammation in both human and rodent models [3,4]. 

Rodent microbiome transplant studies illustrate that dietary change can rapidly alter the gut microbial composition and intestinal mucosal layer for associated health effects including dysbiosis, microbiota encroachment, inflammation, and metabolic disease [5,6,7]. Oral probiotic supplements contain live commensal bacteria and are a potential means to combat these changes. Probiotics containing *Lactobacillus* and *Bifidobacterium* produce anti-inflammatory effects; *Lactobacillus* supplementation reduced circulating levels of pro-inflammatory cytokines in ulcerative colitis patients while *Bifidobacterium infantis* modulated both gut mucosal and systemic inflammation [8,9].

Muscadine grapes (*Vitus rotundifolia*) are rich in polyphenolic compounds including ellagic acid, gallic acid, quercetin, cyanidin, and delphinidin [10]. Muscadine grape extract (MGE) has antibacterial properties and has successfully inhibited pathogenic bacteria (*Helicobacter pylori*) in cell culture [11]. Rodent models of MGE consumption report anti-inflammatory properties in the gut and distal tissues [12]. MGE may be beneficial in modulating diet-associated pathogenesis; clinical MGE supplementation suppressed plasma endotoxin, lipoprotein binding protein, and pro-inflammatory protein expression following a meal high in fat and carbohydrates [13].

We utilize adult female C57BL/6 mice to investigate the beneficial metabolic, microbial, and anti-inflammatory effects of MGE and probiotic intervention in animals consuming WD. We report that the addition of MGE and probiotic interventions to a WD reduced adiposity, inflammation, and fibrosis. These pro-health metabolic outcomes were associated with shifts in the gut microbiome and intestinal health parameters.

## 2. Materials and Methods

### 2.1. Materials

Murine diets (TD.08806 and TD.180300) were purchased from Envigo (Indianapolis, IN, USA). Dako EnVision®+ Dual Link System-HRP (DAB+) staining kits (K4065) were obtained from Agilent (Santa Clara, CA, USA). F4/80 antibody (catalogue #: 70076S) was purchased from Cell Signaling Technologies (Danvers, MA, USA). MCP-1 antibody (catalogue #: ab25124) was obtained from Abcam (Waltham, MA, USA). Sirius red F3B (catalogue #: 36-554-8), saturated aqueous picric acid (catalogue #: P6744), and solid picric acid (catalogue #: 239801) were obtained from Sigma-Aldrich (Burlington, MA, USA). Harris hematoxylin (catalogue #: 1201) and eosin phloxine working solution (catalogue #: 1082) were obtained from Newcomer Supply (Middleton, WI, USA). Alcian Blue (catalogue #: 56735) and Nuclear Fast Red (catalogue #: 56733) were obtained from Abcam (Waltham, MA, USA).

### 2.2. Animal Model

Animal procedures were performed in compliance with IACUC-approved protocols. Healthy and drug-naïve female 7-week-old C57BL/6 mice were purchased from Jackson Laboratory (Bar Harbor, ME, USA) and socially housed in individually ventilated cages with a 12-h light/dark cycle (4 per cage, *n* = 96). At 8 weeks of age, mice were randomized into 12 groups (*n* = 8/group). Mice were administered either CD or WD (Table 1). Mice received diet alone, probiotics (1 × 105 CFU *Lactobacillus acidophilus*, *Lactobacillus plantarum*, *Bifidobacterium bifidum*, *Lactobacillus rhamnosus*, *Lactobacillus bulgaricus*, *Lactobacillus salivarius*, *Lactobacillus brevis*, *Bifidobacterium lactis*, *Lactobacillus paracasei*, and *Lactobacillus casei* suspended in auto-claved water 3 times weekly via oral gavage in housing room), antibiotics (5 mg/mL streptomycin, 1 mg/mL ampicillin, and 1 mg/mL colistin in drinking water), MGE (0.1 phenolics/mL in drinking water), combined MGE and probiotics, or combined MGE and antibiotics. MGE phenolic contents were previously characterized and shown to be high in epicatechin, gallic acid, ellagic acid, procyanidin B2, catechin, and catechin gallate [14]. Diets and intervention strategies were administered for 13 weeks. Body weight was monitored weekly.

At the end of the study, plasma samples were collected and snap-frozen. Visceral adipose tissue (VAT), mammary glands (MG), and intestines were isolated. VAT, liver, and lower right inguinal 4/5 MG were weighed. Liver fat content was determined by EchoMRI. All tissues were fixed in 4% paraformaldehyde overnight and paraffin was embedded. 

### 2.3. Gut Microbial Analysis

DNA isolation and 16S sequencing were performed by Microbiome Insights (Vancouver, BC, Canada). In brief, samples were plated in a MoBio PowerMag Soil DNA Isolation Bead Plate. Fecal DNA extraction was completed using a KingFisher robot according to MoBio’s instructions. Bacterial genes were PCR-amplified using dual-barcoded primers targeting the V4 region of the 16S rRNA genes. Amplicons were sequenced using the Illumina MiSeq system and the 300-bp paired-end kit (v.3). The resulting sequences were denoised and taxonomically classified using the Greengenes (v. 1.39_8) reference database. The Mothur software package (v.1.39.5) was used to identify and cluster OTUs [15].

### 2.4. Immunohistochemistry

MG and VAT slides were stained for F4/80 and MCP-1 immunoreactivity using the Dako DAB+ Staining kit following the manufacturer’s protocol with the addition of a Protein Block solution incubation. Antibodies were diluted 1:100 in Antibody Diluent. Slides were counter-stained with hematoxylin. MG and VAT slides were stained with filtered 0.1% Picrosirius red (PicRed) solution. Hematoxylin and Eosin (H&E) staining of intestines was performed as previously reported [16]. Alcian Blue staining of the intestines was completed following the manufacturer’s protocol.

Microscope imaging was completed using a Mantra microscope. For DAB+, PicRed, and Alcian Blue staining, five representative 20× magnification images were taken with no overlap between image borders. For H&E staining, three images were taken of each slide with no overlap between borders.

DAB+ and PicRed staining were analyzed using InForm image analysis software (version 2.6, Perkin Elmer (Waltham, MA, USA)). F4/80-positive cells were identified using cell segmentation and phenotyping tools. The number of F4/80-positive cells identified with a confidence of 70% or greater was used to calculate macrophages per million pixels. Optical densities of 0.450 and 0.500 were selected as the minimum positive staining for PicRed and MCP-1, respectively. The percent of total positive pixels was calculated.

Alcian Blue quantification was completed manually. The average positive cells per villus was calculated for each image.

H&E staining was quantified using ImageJ software (version 1.53s). The average villus length was calculated for each image. The observable muscularis was separated into thirds across the image and width measurement was taken in each third.

Adipocyte diameter was quantified in ImageJ. Adipocyte diameter was measured across the widest point of the adipocyte. The average of the three representative adipocytes was calculated for each image.

### 2.5. RT-PCR

RNA was isolated from snap-frozen liver tissue using the Trizol reagent according to the manufacturer’s protocol (Invitrogen (Carlsbad, CA, USA), reference number 15596018). RNA concentration was measured with a Bio-Rad SmartSpec 3000. cDNA was synthesized from 5 µg of total RNA using Superscript first strand RT-PCR reagents (Thermo Fisher (Waltham, MA, USA), catalogue #: 12574026). RT-PCR was then performed using SYBR green real-time PCR Master Mix (Applied Biosystems (Foster City, CA, USA), catalogue #: A25777) with specific primers for CD68 (F. ACCGCCATGTAGTCCAGGTA, R. ATCCCCACCTGTCTCTCTCA), IL-1 alpha (F. TCTATGATGCAAGCTATGGCTCA, R. CGGCTCTCCTTGAAGGTGA), and TNF alpha (F. GGTCTGGGCCATAGAACTGA, R. CAGCCTCTTCTCATTCCTGC). HPRT (F. CATAACCTGGTTCATCATCGC, R. TCCTCCTCAGACCGCTTTT) served as an internal control. Results were quantified as cycle threshold (Ct) values and expressed as the ratio of target/control (relative Gene Expression to HPRT) using the 2^−ΔΔCt^ method.

### 2.6. Statistical Analysis

Body weight, VAT weight, mammary gland weight, liver weight, liver adiposity, Shannon Diversity Index, and proportional abundance of bacterial taxa were compared using one-way ANOVA with Tukey’s multiple comparisons test. To compare bacterial β-diversity, Principal Component analysis was performed using an Adonis test with a post-hoc pairwise test with FDR correction for multiple comparisons. Immunohistochemistry data was analyzed by grouping images from each animal and completing a main effects two-way ANOVA and Tukey’s multiple comparisons test. Graphs are plotted with standard error bars.

## 3. Results

**Figure 1:**
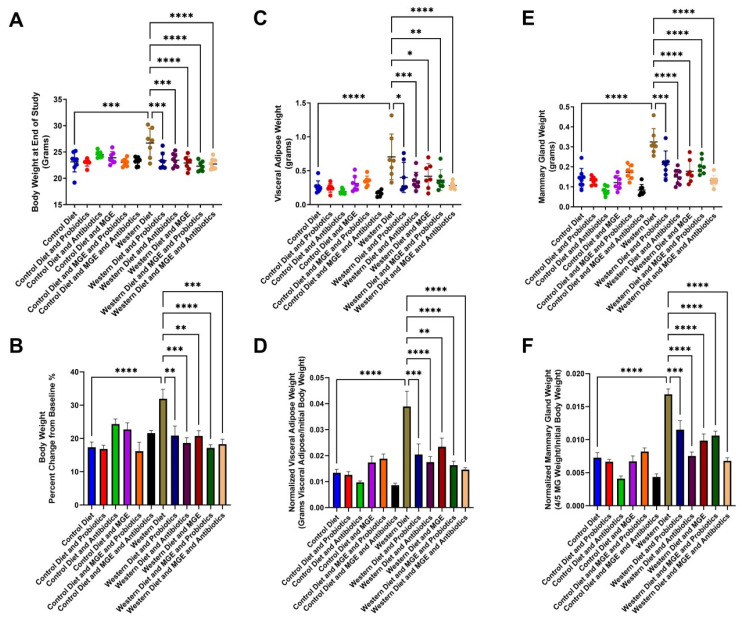
Dietary intervention modulates body condition and tissue morphology. (**A**) Body weight of female C57BL/6 mice following 13 weeks of exposure to diets and intervention strategies. (**B**) % change in body weight. (**C**) Visceral adipose tissue weight at study completion. (**D**) Normalized visceral adipose tissue weight at the end of the study. (**E**) Weight of the right lower (4/5) mammary gland at study completion. (**F**) Normalized inguinal mammary gland weight. *n* = 7–8. * *p* < 0.05, ** *p* < 0.01, *** *p* < 0.001, **** *p* < 0.0001.

Dietary intervention modulates body condition and tissue morphology. At the end of the study, WD-fed mice had a 1.15-fold increased body weight compared to CD (*p* = 0.0001, Figure 1A). Mice consuming WD undergoing interventions with probiotics, antibiotics, MGE, combined MGE and probiotics, and MGE and antibiotics displayed significantly reduced body weight compared to WD alone (Figure 1A). Percent change in body weight is shown in Figure 1B. WD-fed mice had an average 2.66-fold increase in visceral adipose tissue weight compared to CD (*p* < 0.0001, Figure 1C). In WD-fed animals, visceral adiposity was reduced by all interventions. Visceral adipose tissue weight was normalized to initial body weight (Figure 1D). Compared to CD-fed mice, mammary glands from WD-fed animals were 2.27-fold heavier (*p* < 0.0001, Figure 1E). The average mammary gland weight of WD-fed animals was reduced by probiotics, antibiotics, MGE, MGE and probiotics, and by MGE and antibiotics interventions (*p* < 0.0001). Inguinal mammary gland weight was normalized to initial body weight (Figure 1F).

Dietary intervention strategies modulate liver adiposity and glucose metabolism in WD-fed mice. CD-fed mice consuming MGE and antibiotics had reduced liver weight compared to diet alone (*p* = 0.0032, Figure S1A). WD-fed mice receiving probiotics, combined MGE and antibiotics, or combined MGE and probiotics had reduced liver weight compared to WD alone (*p* = 0.0225, *p* = 0.006, *p* = 0.006). Compared to WD-fed animals, those receiving probiotics (alone or combined with MGE) had reduced liver adiposity (*p* < 0.0001, Figure S1B). Combined MGE and probiotic intervention was associated with lower liver adiposity than MGE alone (*p* = 0.002). We also measured inflammatory gene expression in the livers of mice. A combination of MGE and probiotics reduced liver TNFα gene expression in both CD and WD-fed animals (Figure S1C). In WD-fed animals, administration of antibiotics, MGE, a combination of MGE and probiotics, and MGE + antibiotics reduced liver IL-1α gene expression (Figure S1D). WD consumption increased liver CD68 expression compared to livers of CD-fed mice. All interventions in WD-fed animals reduced liver CD68 gene expression (Figure S1E). WD-fed mice had higher glucose tolerance test areas under the curve compared to CD animals (*p* = 0.005). WD-fed mice displayed elevated area under the curve compared with all CD-fed mice except those receiving antibiotics (Figure S2A,B).

**Figure 2:**
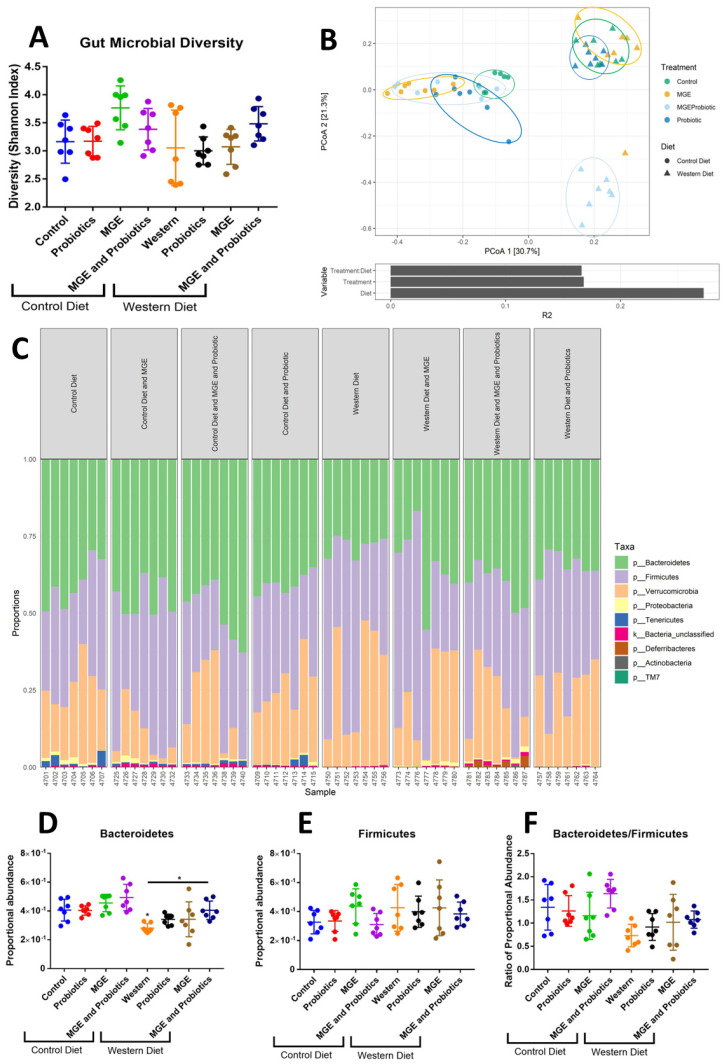
Diet composition and intervention strategies modulate gut microbiome composition. (**A**) Shannon diversity index of fecal samples collected after 13 weeks of diet and intervention exposure. (**B**) Principal component analysis of fecal microbial composition. (**C**) Proportional abundance of bacterial phyla identified in fecal samples. Each bar represents data collected from one mouse. (**D**) Proportional abundance of fecal Bacteroidetes. (**E**) Proportional abundance of fecal Firmicutes. (**F**) The ratio of Bacteroidetes to Firmicutes. *n* = 7–8, * *p* < 0.05.

Diet composition and intervention strategies modulate gut microbiome composition. The Shannon α-diversity index of fecal bacteria did not significantly vary between groups (Figure 2A). An Adonis test determined that Treatment, Diet, and Treatment: Diet interactions all significantly impacted gut bacterial beta diversity with R^2^ values of 0.1681, 0.2724, and 0.1661, respectively (*p* = 0.0001, Figure 2B). Post-hoc pairwise testing demonstrated that CD-fed mice receiving probiotics, MGE, and combined MGE and probiotics all significantly varied from animals receiving CD alone (*p* = 0.004, *p* = 0.002, *p* = 0.003). CD-fed mice receiving either MGE or probiotics alone expressed different gut bacterial contents than those receiving combined MGE and probiotic intervention (*p* = 0.024, *p* = 0.002). WD-fed mice were significantly different from CD (*p* = 0.002). In WD-fed mice, those receiving combined intervention with MGE and probiotics expressed a gut bacterial diversity different from those consuming chow alone, probiotics, or MGE (*p* = 0.003, *p* = 0.002, *p* = 0.002). Observed bacterial phyla are illustrated in a bar graph (Figure 2C). The proportional abundance of the Bacteroidetes and Firmicutes phyla were compared (Figure 2D–F). WD-fed mice had 1.44-fold lower Bacteroidetes abundance than CD (*p* = 0.0349, Figure 2D). Combined MGE and probiotic intervention of WD-fed animals prevented this effect (*p* = 0.0381). No significant diet and intervention-mediated effects on Firmicutes abundance or Bacteroidetes/Firmicutes ratio were observed (Figure 2E,F).

**Figure 3:**
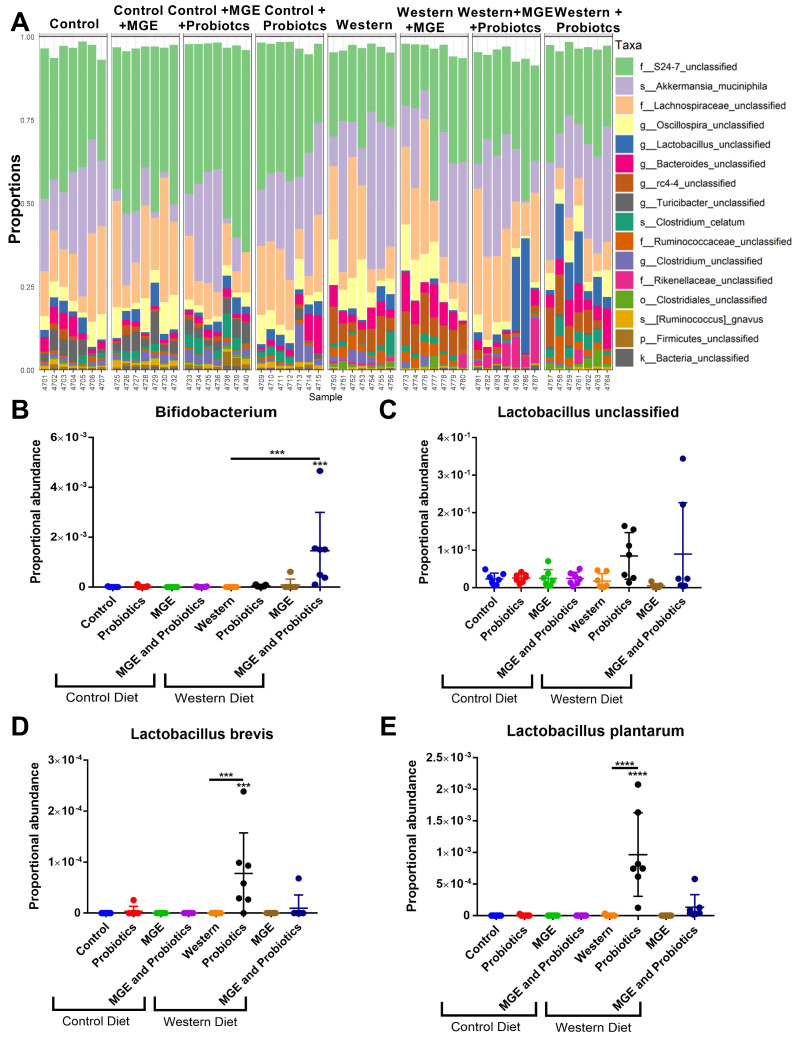
Dietary intake and MGE consumption mediate gut colonization of probiotic bacterial species. (**A**) Proportional abundance of bacterial species were identified in murine feces following 13 weeks of diet and intervention exposure. Each bar represents the fecal bacterial composition of a single mouse. (**B**–**E**) Proportional abundance of probiotic bacterial species identified in murine feces. (**B**) *Bifidobacterium*. (**C**) *Lactobacillus unclassified*. (**D***) Lactobacillus brevis*. (**E**) *Lactobacillus plantarum*. *** *p* < 0.001. **** *p* < 0.0001.

Dietary intake and MGE consumption mediate gut colonization of probiotic bacterial species. The bacterial species’ proportional abundance is illustrated in a bar graph (Figure 3A). *Bifidobacterium* were enriched in WD-fed mice receiving combined intervention with MGE and probiotics compared to those receiving diet alone (proportional abundance 0.001457 vs. 0, *p* = 0.002, Figure 3B). The proportional abundance of unclassified *Lactobacillus* species was not significantly altered between groups (*p* > 0.2, Figure 3C). *Lactobacillus brevis* was not observed in samples from WD or CD-fed mice but had an average proportional abundance of 7.784 × 10^−5^ in WD-fed mice receiving probiotics (*p* = 0.0003, Figure 3D). WD-fed animals receiving probiotics expressed a 215-fold increase in fecal *Lactobacillus plantarum* compared to diet alone (*p* < 0.0001, Figure 3E).

Diet and intervention strategies mediate gut colonization of disease-associated microbes. *Lactococcus* were enriched 1810-fold in WD-fed mice compared to CD (*p* < 0.0001, Figure S3A). In WD-fed animals, MGE reduced this effect while combined intervention was associated with reduced *Lactococcus* (*p* = 0.0269, *p* = 0.0213). *Ruminococcaceae* was reduced in WD-fed mice consuming combined MGE and probiotics compared to diet alone (Figure S3B). *Anaerovorax* were observed in WD-fed mice but not in mice receiving MGE (alone or combined with probiotics) or CD (*p* = 0.0049, Figure S3C). *Alistipes* were enriched in WD-fed mice with combined MGE and probiotics compared to WD (Figure S3D). Tukey multiple comparison analysis did not identify differences in *Oscillospira* enrichment between groups, but ANOVA analysis of the entire dataset suggests a trend (*p* = 0.0127, Figure S3E). *Enterococcus* was enriched in WD-fed mice compared to CD (*p* = 0.0003, Figure S3F). Probiotics reduced this effect while combined intervention yielded a reduction in WD-fed animals (*p* = 0.008, *p* = 0.0012).

**Figure 4:**
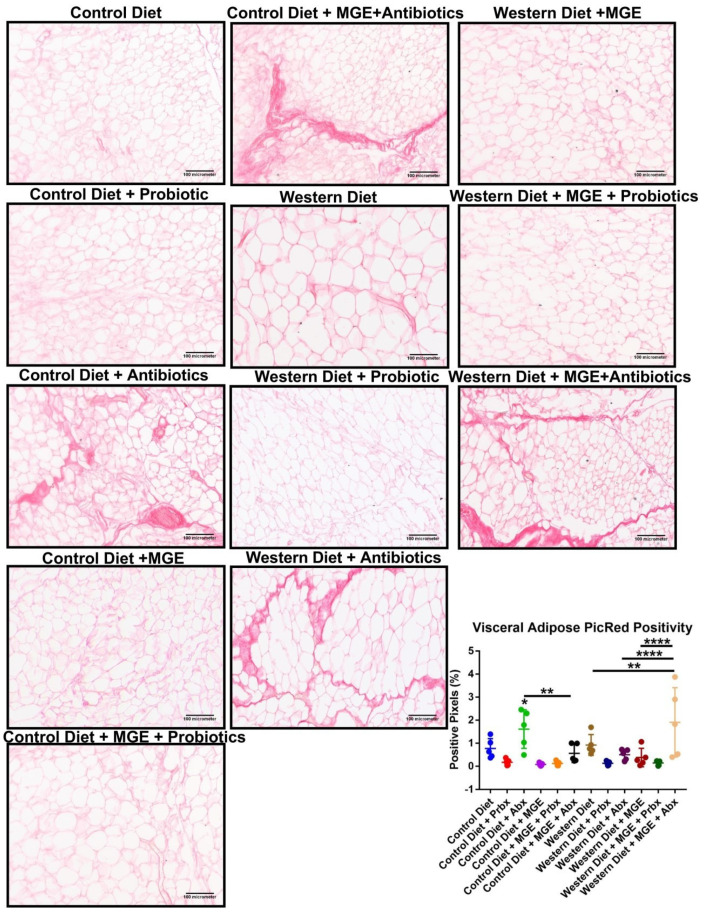
Diet and intervention strategies mediate changes in visceral adipose tissue fibrosis. Representative 20× images of visceral adipose tissue stained with Picrosirius Red. Percentage of pixels positive for PicRed staining. * *p* < 0.05. ** *p* < 0.01. **** *p* < 0.0001.

**Figure 5:**
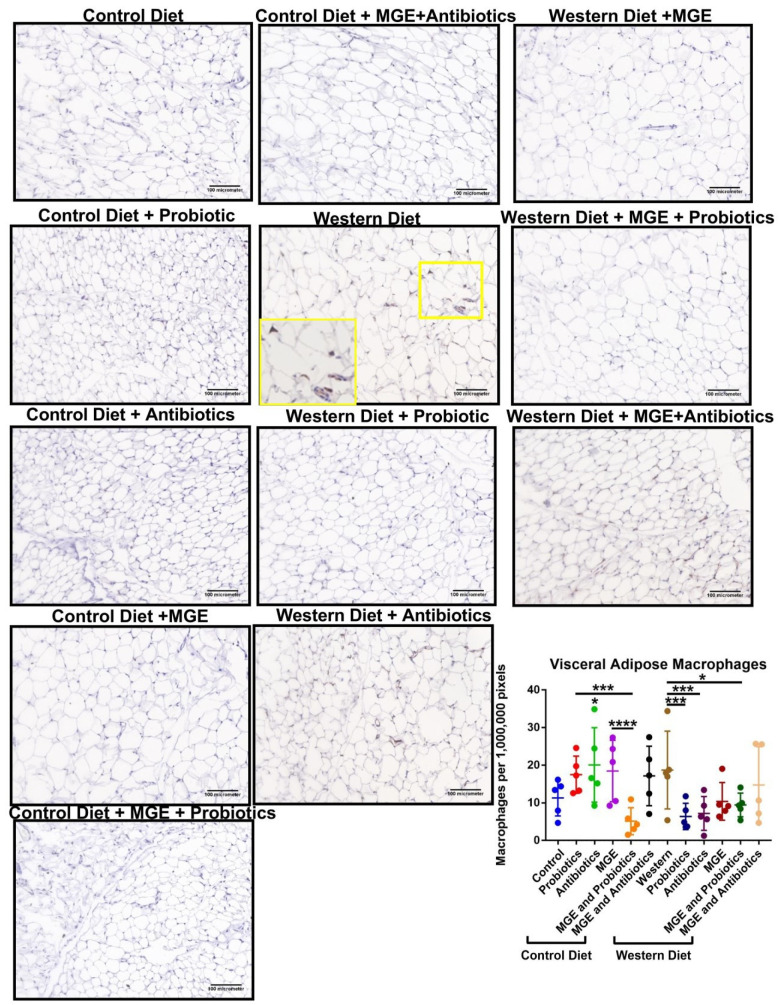
Diet and intervention strategies mediate changes in visceral adipose tissue immune cell infiltration. Representative 20× images of visceral adipose tissue stained with anti-F4/80. Number of F4/80-positive macrophages identified per million pixels. * *p* < 0.05. *** *p* < 0.001. **** *p* < 0.0001.

**Figure 6:**
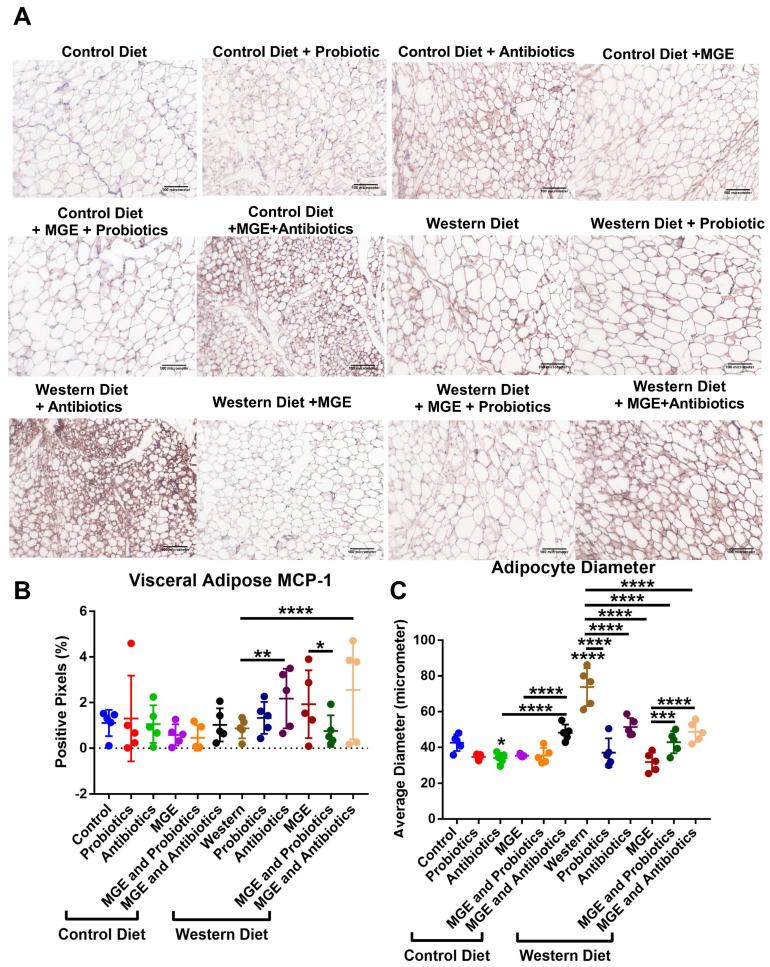
Diet and intervention strategies mediate changes in visceral adipose tissue physiology and inflammation. (**A**) Representative 20× images of visceral adipose tissue stained with anti-MCP-1. (**B**) Percentage of pixels positive for anti-MCP-1 staining. (**C**) Average adipocyte diameter calculated from three representative adipocytes per image. * *p* < 0.05. ** *p* < 0.01. *** *p* < 0.001. **** *p* < 0.0001.

Diet and intervention strategies mediate changes in visceral adipose tissue physiology. VAT was stained with Picrosirius Red (a pan-collagen stain to determine tissue fibrosis (Figure 4)) or antibodies to F4/80 (a pan-macrophage marker to determine immune cell recruitment to the tissue (Figure 5) and MCP-1 (a monocyte-attracting chemokine as a marker for tissue inflammation (Figure 6). CD-fed mice receiving antibiotics had an average PicRed positivity 2.09-fold greater than diet alone (*p* = 0.0439, Figure 4). The addition of MGE to mice consuming antibiotic water was associated with a 2.87-fold reduction of this effect (*p* = 0.0024). In WD-fed mice, combined MGE and antibiotics yielded a 2.06-fold increase compared to diet alone (*p* = 0.0067). Comparatively, those receiving either antibiotics or MGE alone had respective 3.80 and 5.00-fold reduced PicRed positivity (*p* < 0.0001). CD-fed mice receiving antibiotics had an average 1.77-fold increase in macrophages compared to CD (*p* = 0.0422, Figure 5). CD-fed mice receiving combined MGE and probiotics had an average macrophage count 3.42-fold lower than probiotics alone and 3.61-fold lower than MGE alone (*p* = 0.0002, *p* < 0.0001). Macrophages in WD-fed mice receiving probiotics, antibiotics, and combined MGE and probiotics were reduced 2.93, 2.60, and 1.98-fold, respectively, compared to diet alone (*p* = 0.0003, *p* = 0.0009, *p* = 0.0228). WD-fed mice consuming antibiotics and combined MGE and antibiotics had an average of 2.50 and 2.93-fold increases in MCP-1 positivity compared to diet alone (*p* = 0.0066, *p* < 0.0001, Figure 6B). Compared to WD-fed mice receiving MGE alone, those consuming MGE combined with probiotics had an average 2.56-fold lower MCP-1 positivity (*p* = 0.0248). CD-fed mice receiving antibiotics had an average 1.26-fold reduced adipocyte diameter than diet alone (*p* = 0.0226, Figure 6C). Mice receiving combined MGE and antibiotics had 1.42 and 1.36-fold higher average adipocyte diameter compared to antibiotics or MGE alone (*p* < 0.0001). WD-fed mice had a 1.73-fold greater adipocyte diameter than CD (*p* < 0.0001). This effect was reduced by probiotics, antibiotics, MGE, combined MGE and probiotics, and combined MGE and antibiotics by respective magnitudes of 1.99, 1.44, 2.32, 1.72, and 1.51-fold (*p* < 0.0001). Consumption of MGE alone in WD-fed mice resulted in an average adipocyte diameter of 1.35 and 1.53-fold greater than combined intervention with MGE and probiotics or MGE and antibiotics (*p* = 0.0006, *p* < 0.0001).

Diet and intervention strategies mediate factors associated with mammary gland fibrosis and inflammation. MG were stained with PicRed, F4/80, and MCP-1 (Figure S5A). In CD-fed mice, probiotics and combined MGE and probiotics reduced PicRed positivity 2.63 and 2.16-fold, respectively (*p* = 0.0021, *p* = 0.0166, Figure S5B). Compared to mice receiving WD alone, mammary gland PicRed positivity was reduced 3.51-fold by probiotics, 2.04-fold by MGE, and 2.46-fold by combined MGE and probiotics (*p* = 3.51, *p* = 2.04, *p* = 2.46). Compared to CD alone, the number of macrophages per million pixels increased 1.73 and 2.21-fold in mice receiving MGE and combined MGE and antibiotics, respectively (*p* = 0.0439, *p* < 0.0001, Figure S5C). Mice receiving combined MGE and antibiotic intervention had an average macrophage count 2.38-fold higher than antibiotic intervention alone (*p* < 0.0001). WD-fed mice receiving antibiotics had 1.92-fold more macrophages than those consuming WD alone (*p* < 0.0001). WD-fed mice receiving combined MGE and antibiotics had 1.85-fold fewer macrophages than antibiotics alone (*p* < 0.0001). MCP-1 positivity was elevated in CD-fed mice receiving antibiotics and combined MGE and antibiotic intervention compared to diet alone by respective magnitudes of 48.63 and 31.76-fold (*p* < 0.0001, *p* = 0.0002, Figure S5D). Antibiotic intervention of WD-fed animals was associated with a 3.53-fold increased MCP-1 positivity compared to diet alone, while combined antibiotic and MGE intervention yielded a 2.16-fold reduction compared to antibiotics (*p* < 0.0001).

**Figure 7:**
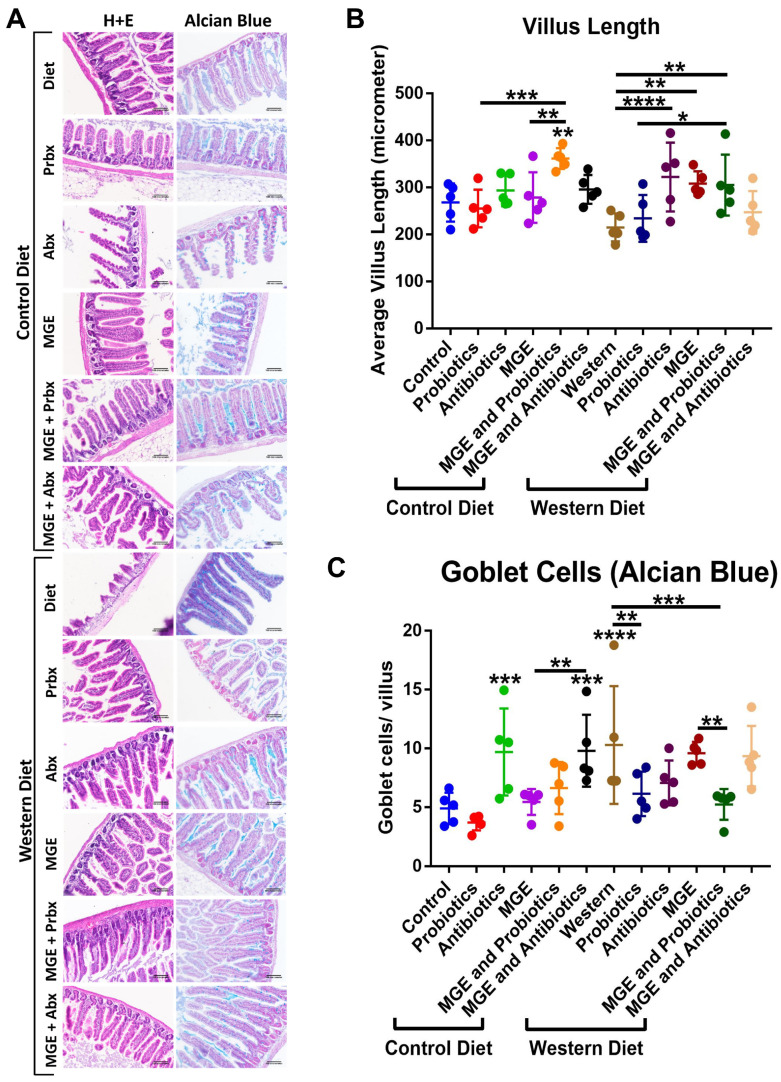
Diet and intervention strategies mediate factors associated with intestinal inflammation. (**A**) Images of intestinal sections stained with H&E and Alcian Blue. (**B**) Average villus length measured in H&E images. (**C**) Alcian Blue-positive goblet cells counted per villus. * *p* < 0.05. ** *p* < 0.01. *** *p* < 0.001. **** *p* < 0.0001.

Diet and intervention strategies mediate factors associated with intestinal inflammation. Intestines were stained with H&E and Alcian Blue (Figure 7A). CD-fed mice receiving combined MGE and probiotics had an average villus length 1.35, 1.41, and 1.30-fold higher than those receiving diet alone, probiotics, and MGE respectively (*p* = 0.0012, *p* = 0.0001, *p* = 0.0072 Figure 7B). WD-fed mice receiving antibiotics, MGE, and combined MGE and probiotics had average villus lengths greater than diet alone by magnitudes of 1.50, 1.43, and 1.42-fold, respectively (*p* < 0.0001, *p* = 0.0012, *p* = 0.0021). WD-fed mice receiving combined MGE and probiotics had an average villus length 1.30-fold greater than probiotics alone (*p* = 0.0439). We did not observe a significant impact of diet or intervention strategy on muscularis width (Supplemental Figure S6). CD-fed mice receiving antibiotics (alone or combined with MGE) had 1.98 and 2.00-fold more goblet cells per villus compared to CD (*p* = 0.0009, *p* = 0.0006, Figure 7C). Intestines of mice receiving combined MGE and antibiotics had 1.80-fold more goblet cells/villus than MGE alone (*p* = 0.0041). WD-fed mice had 2.10-fold more goblet cells per villus than CD-fed animals (*p* < 0.0001). WD-fed mice receiving intervention with probiotics and combined MGE and probiotics had 1.68 and 1.97-fold reduced goblet cells/villus compared to WD alone (*p* = 0.0082, *p* = 0.0003). Combined MGE and probiotic intervention was associated with a goblet cell count 1.83-fold lower than MGE alone (*p* = 0.0041).

## 4. Discussion

Clinical dietary polyphenol interventions report anti-inflammatory and antioxidant properties [17]. *Lactobacillus*-based probiotics reduced inflammation, glucose intolerance, and insulin resistance in a study using a male murine model of diet-induced obesity [18]. These models suggest polyphenols and probiotics as potential strategies for the modulation of WD-associated inflammatory, microbial, and metabolic effects. We investigated a unique combination of a *Lactobacillus* and *Bifidobacterium* probiotic with an extract high in dietary polyphenols (MGE) for the intervention of WD-fed female mice.

The increase in WD-mediated body weight and visceral adipose were alleviated by dietary intervention. High visceral adipose tissue deposition in obese patients is associated with cardiometabolic disease risk [19]. MGE combined with probiotics reduced body weight and all interventional strategies reduced visceral adiposity in WD-fed mice. Muscadine grape phytochemical supplementation of high-fat diet-fed male C57BL/6 mice was associated with a 12% body weight reduction compared to diet alone [20]. Clinical models report beneficial effects of *Lactobacillus*-based probiotic supplementation on weight loss [21]. A trial of obese subjects supplemented with *Lactobacillus rhamnosus* demonstrated that probiotic-mediated weight loss and fat mass reduction were greater in women than men [22]. Antibiotic-mediated gut microbiome depletion contributed to visceral adipose tissue browning in high-fat diet-fed male mice [23]. While we did not measure brown adipose tissue in this study, these effects may have contributed to the observed perturbations in visceral adiposity in WD-fed mice receiving antibiotics in our model. While sex differences have been reported in body weight gain and lipid accumulation, many of the available dietary intervention models rely on male animals. Our findings contribute important data regarding female responses to dietary intervention.

We previously reported that WD was associated with reduced gut bacterial α-diversity compared to Mediterranean Diet in a nonhuman primate (NHP) model [24]. We did not observe significant variation in gut microbial alpha diversity in our model, but we report that diet, intervention, and diet + intervention all significantly impacted β-diversity. Our NHP model demonstrated that diet (Western or Mediterranean) was the main determinant of gut microbial β-diversity [24], but our observation of the impact of intervention strategy (probiotics, MGE, and antibiotics) on gut β-diversity is a novel finding. We examined the bacterial phyla abundance present in the gut. Bacteroidetes and Firmicutes are the most abundant bacterial phyla observed in human adults [25]. Reduced gut Bacteroidetes are associated with obesity [26]. WD-fed mice echoed this response and combined MGE and probiotic intervention was associated with increased Bacteroidetes in WD-fed mice. A study of male Wistar rats consuming a high-fat diet reported that the diet was associated with reduced Bacteroidetes but animals receiving polyphenol intervention (quercetin and resveratrol) did not significantly differ from CD [27]. Clinical intervention with *Lactobacillus*-based probiotics in inflammatory bowel disease patients restored Bacteroidetes to normal levels [28]. We report that neither MGE nor our administered probiotic altered Bacteroidetes colonization in WD-fed animals alone, but the combined intervention may have an additive effect. Gut enrichment of Firmicutes is associated with obesity and a low Bacteroidetes/Firmicutes ratio indicates an obesity-favoring gut microbial population [29]. We did not observe significant alteration in Firmicutes abundance or the Bacteroidetes/Firmicutes ratio. In the previously discussed rat model of polyphenol intervention, the supplement was associated with reduced gut Firmicutes compared to high-fat diet-fed animals [27]. Our administered dietary polyphenol solution (MGE) is low in both resveratrol and quercetin but does contain catechin. An in vitro model of cultured fecal samples supplemented with polyphenols demonstrated that catechin inhibited both Bacteroidetes and Firmicutes growth and was associated with reduced energy metabolism [30]. This effect may explain why MGE alone did not increase Bacteroidetes colonization as well as the resulting insignificant Bacteroidetes/Firmicutes ratios.

We examined the abundance of fecal probiotic bacteria. *Bifidobacterium*, *Lactobacillus unclassified*, *Lactobacillus brevis*, and *Lactobacillus plantarum* were only observed in the fecal microbial contents of WD-fed mice. *Bifidobacterium* was enriched in animals receiving combined MGE and probiotics while *Lactobacillus brevis* and *Lactobacillus plantarum* were enriched in those receiving probiotics alone. When *Lactobacillus* and *Bifidobacterium* were supplemented in vitro with either grape seed extract, monomeric-rich grapeseed extract, or oligomeric-rich grapeseed extract, *Bifidobacterium* reached maximal growth with the monomeric-rich and oligomeric-rich varieties and not with whole extract [31]. These results suggest that MGE-mediated gut bacterial colonization changes with the composition of the specific extract being administered.

Adipose tissue fibrosis is an obesity-associated response in which extracellular matrix deposition accumulates collagen fibers that trap adipocytes, reducing tissue plasticity [19]. Mature adipocytes cultured with decellularized material from obese patient adipose tissue indicated that adipose tissue fibrosis may result in reduced adipokine secretion, decreased lipolysis, and increased pro-inflammatory cytokine secretion [32]. In WD-fed mice, combined MGE and antibiotics were associated with elevated collagen deposition compared to WD alone, MGE alone, or antibiotics alone. Adipose tissue inflammation is associated with obesity-mediated insulin resistance in rodents [33,34]. This is largely macrophage-mediated and macrophage accumulation has been identified in the adipose tissue of obese humans [35]. WD-fed mice receiving probiotics, antibiotics, and combined MGE and probiotics had fewer visceral adipose macrophages compared to diet alone. MCP-1 is a chemotactic factor for monocytes that is primarily produced by epithelial cells and macrophages [36]. MCP-1 mRNA was enriched in the adipose tissue and plasma of mice with high-fat diet-induced obesity [36]. We anticipated that WD intake would result in increased MCP-1, but we did not observe this effect. Visceral adipose MCP-1 was elevated in WD-fed mice receiving antibiotics (both alone and with MGE), linking VAT fibrosis and inflammation. The gut microbiome can have immunomodulatory effects [37]. It is possible that microbiome depletion removed protective effects against WD intake, allowing an MCP-1-mediated inflammatory response. Adipocyte hypertrophy (enlarged adipocyte size) results in increased secretion and expression of pro-inflammatory cytokines [19,34]. In CD-fed mice, antibiotics were associated with reduced adipocyte diameter while combined MGE and antibiotics ameliorated this effect. WD-fed mice receiving antibiotics (either alone or with MGE) had reduced adipocyte size compared to diet alone. These results corroborate our fibrosis staining, as antibiotics were associated with a fibrotic response. Excessive collagen deposition in the adipose tissue prevents adipocytes from expanding [19]. WD-fed mice displayed adipocyte hypertrophy compared to controls, while probiotics, MGE, and combined MGE and probiotics reduced this effect. *Lactobacillus paracasei* ameliorated lipid accumulation in the epididymal white adipose tissue of high-fat diet-fed mice, preventing adipocyte hypertrophy [38]. In vitro study of polyphenol-treated 3T3-L1 adipocytes resulted in reduced adipocyte triglyceride content and down-regulation of lipogenic genes, suggesting a potential metabolically beneficial response [39].

Elevated breast volume is associated with high BMI [40]. MG weight was significantly increased in mice consuming WD. We report a novel effect of dietary intervention strategies (antibiotics, MGE, MGE and probiotics, and MGE and antibiotics) in reducing WD-mediated MG weight. As many dietary intervention studies have utilized male rodents, MG weight has not been reported. These results concur with available knowledge regarding dietary intervention and adiposity; however, further study examining MG will be beneficial. MG fibrosis contributes to increased breast density which is correlated with breast cancer risk [41]. Intervention with probiotics, MGE, and combined MGE and probiotics reduced MG collagen deposition in WD-fed mice, suggesting that oral probiotics and polyphenol administration could modulate diet-associated breast fibrosis. Damaged inflammatory homeostasis in obese patients can contribute to a feed-forward loop upregulating macrophage-mediated inflammation in the mammary tissue [42]. CD-fed mice receiving both MGE and combined MGE and antibiotic intervention had more MG macrophages than controls. In WD-fed animals, antibiotics were associated with a larger macrophage population compared to diet alone. MG tissue maintains a population of resident macrophages that function in tissue development and remodeling [43]. MCP-1 was elevated in CD-fed mice receiving both antibiotics and combined MGE and antibiotics, while in WD-fed mice the effect was observed in those receiving antibiotics alone. Elevated MG MCP-1 was associated with increased MG density and breast cancer risk in a murine model [44]. We observed MCP-1 enrichment in animals with depleted gut microbiota, which suggests a novel effect of the gut microbiome in protecting mammary tissue from WD-mediated fibrosis.

Intestinal inflammation is associated with reduced villus length and muscularis thickening [45,46]. Combined MGE and probiotic intervention increased villus length in mice. This effect was greater in mice receiving combined intervention than in those receiving MGE or probiotics on CD or probiotics on WD. Oral polyphenols alleviated colitis symptoms by modulating the gut microbiome in a murine model [47]. Probiotic administration yielded immunomodulatory effects on murine colitis [48]. The number of goblet cells increases from the duodenum to the distal colon, but microbiota also plays a role as germ-free mice have fewer goblet cells than wild-type mice [49]. Antibiotic interventions were associated with a significant increase in goblet cells of CD-fed mice, identifying a difference between antibiotic microbiome depletion and germ-free mice. WD-fed mice had more goblet cells than control mice which was modulated by probiotic intervention. This suggests that gut microbial contents influence goblet cell count, a concept corroborated by germ-free mouse goblet cell reduction [50].

## 5. Conclusions

In conclusion, our study presents a novel dietary intervention strategy combining commensal probiotics and polyphenol-enriched MGE to induce metabolic, microbial, and immunomodulatory shifts in WD-fed mice. We utilized female mice, which are often not included in obesity dietary intervention models. Our study is limited by this factor. Future studies are needed to determine the exact molecular mechanisms connecting observed gut microbial shifts to the beneficial effects observed in the VAT, MG, and intestinal tissues.

## Figures and Tables

**Table 1:** The nutritional composition of murine chow diets.

	CD	WD
	(TD.08806)	(TD.180300)
Protein (% kcal)	20.5%	15.9%
Carbohydrates (% kcal)	69.1%	39.6%
Fat (% kcal)	10%	44.5%
Saturated Fat	27%	43.3%
Monounsaturated Fat	36.5%	35.1%
Polyunsaturated Fat	36.5%	20.5%
Sucrose	11.2%	25.5%
Cholesterol (mg/kg)	0.017	0.056
Sodium (g/kg)	0.28	1.59

## Data Availability

All data is contained within the manuscript and Supplementary Figures.

## Supplementary Materials

The following supporting information can be downloaded at:https://www.mdpi.com/article/10.3390/cells12222599/s1, Figure S1: Dietary intervention strategies modulate liver adiposity in Western diet-fed mice; Figure S2: Diet and antibiotic administration modulate glucose homeostasis; Figure S3: Diet and intervention strategies mediate gut colonization of disease-associated microbes; Figure S4: IHC negative controls; Figure S5: Diet and intervention strategies mediate factors associated with mammary gland fibrosis and inflammation; Figure S6: Average intestinal muscularis thickness calculated from measurements of the H&E images.
