# Supplementary material for: Probiotic and Muscadine Grape Extract Interventions Shift the Gut Microbiome and Improve Metabolic Parameters in Female C57BL/6 Mice
Source: Cells. 2023 Nov 10;12(22):2599. doi: 10.3390/cells12222599 (PMC10670540; doi:10.3390/cells12222599)

Supplemental Figure S1. Dietary intervention strategies modulate liver adiposity in Western diet-fed mice. (A) Liver weight at study completion. (B) Liver adiposity as determined by EchoMRI. (C) Liver TNF-alpha gene expression as determined by RT-PCR. (D)Liver IL-1 alpha gene expression as determined by RT-PCR. (E) Liver CD68 gene expression as determined by RT-PCR. *p<0.05, **p<0.01, ***p<0.001, ****p<0.0001.

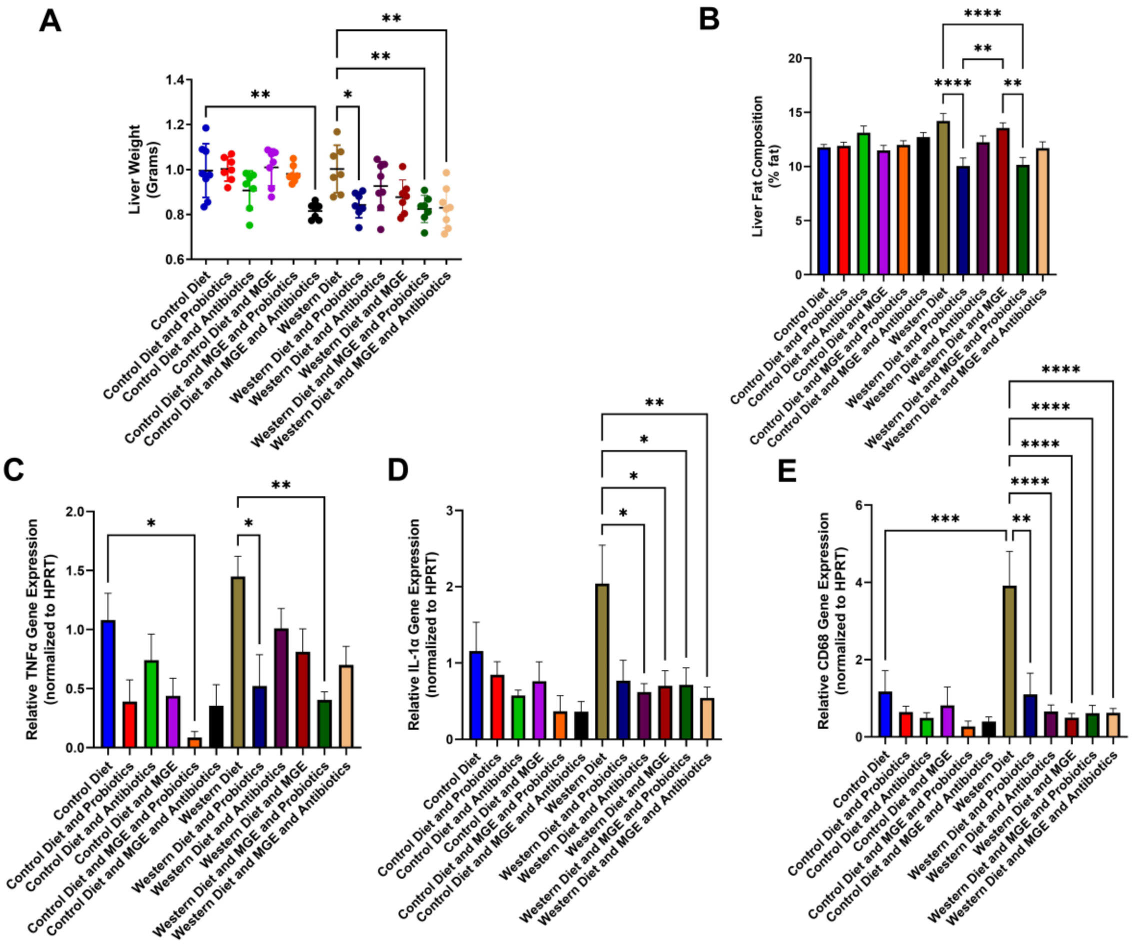

Supplemental Figure S2. Diet and antibiotic administration modulate glucose homeostasis. A) Average blood glucose over time following overnight fasting and intraperitoneal injection with 10 mg/kg glucose solution. Each line represents the average blood glucose of the entire group (n=8). (D) Area under the curve of average blood glucose over time for each individual animal. *p<0.05, **p<0.01, ***p<0.001, ****p<0.0001.

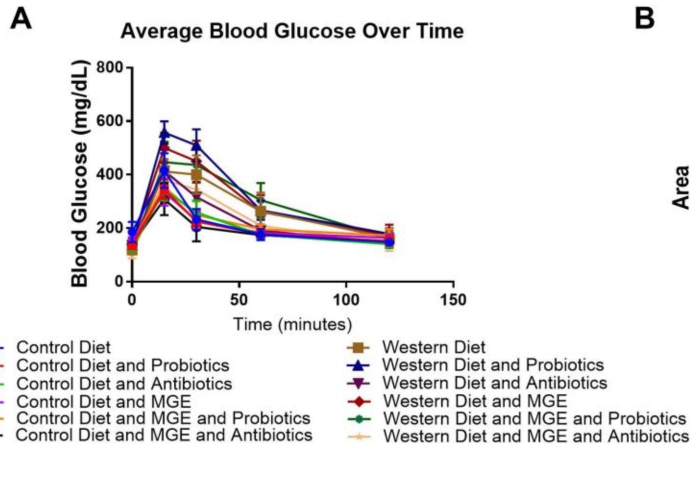

Supplemental Figure S3. Diet and intervention strategies mediate gut colonization of disease-associated microbes. Fecal proportional abundance of (A) Lactococcus, (B) Ruminococcaceae, (C) Anaerovorax, (D) Alistipes, (E) Oscillospira, and (F) Enterococcus. *p<0.05. **p<0.01. ***p<0.001. ****p<0.0001.

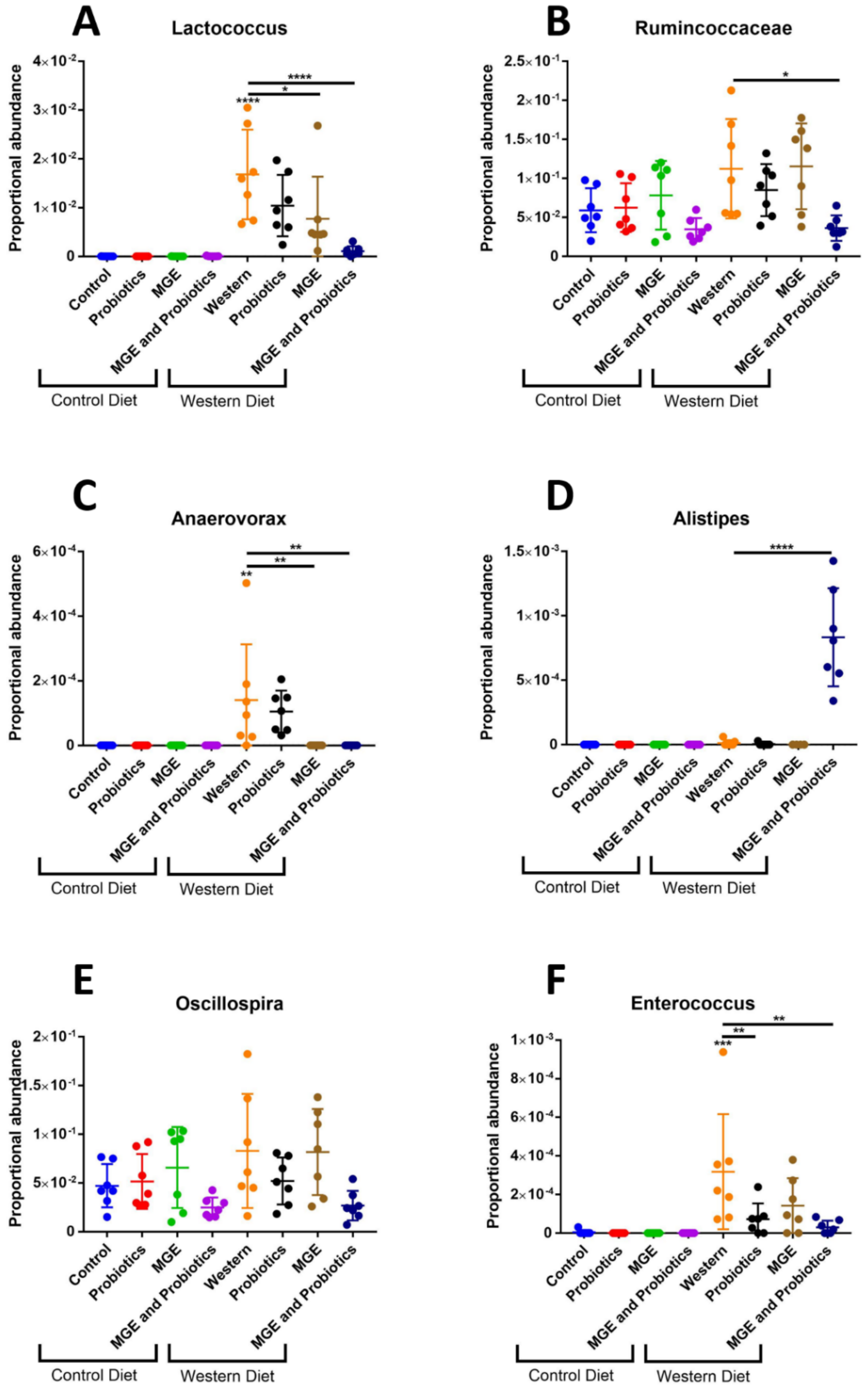

Supplemental Figure S4. IHC negative controls.

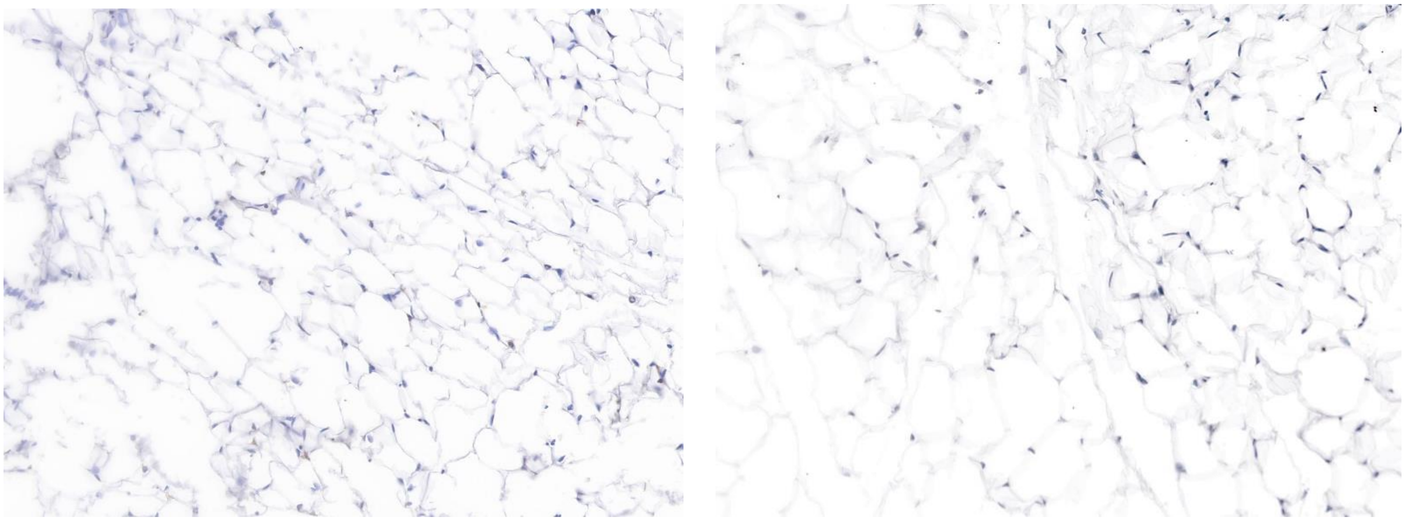

Supplemental Figure S5: Diet and intervention strategies mediate factors associated with mammary gland fibrosis and inflammation. (A) Images of mammary glands stained with PicRed, anti-F4/80, and anti-MCP-1 at 20x magnification. (B) Percentage of pixels positive for PicRed staining. (C) Number of F4/80-positive macrophages identified per million pixels. (D) Percentage of pixels positive for anti-MCP-1 staining. ***p<0.05. **p<0.01. ***p<0.001. ****p<0.0001.**

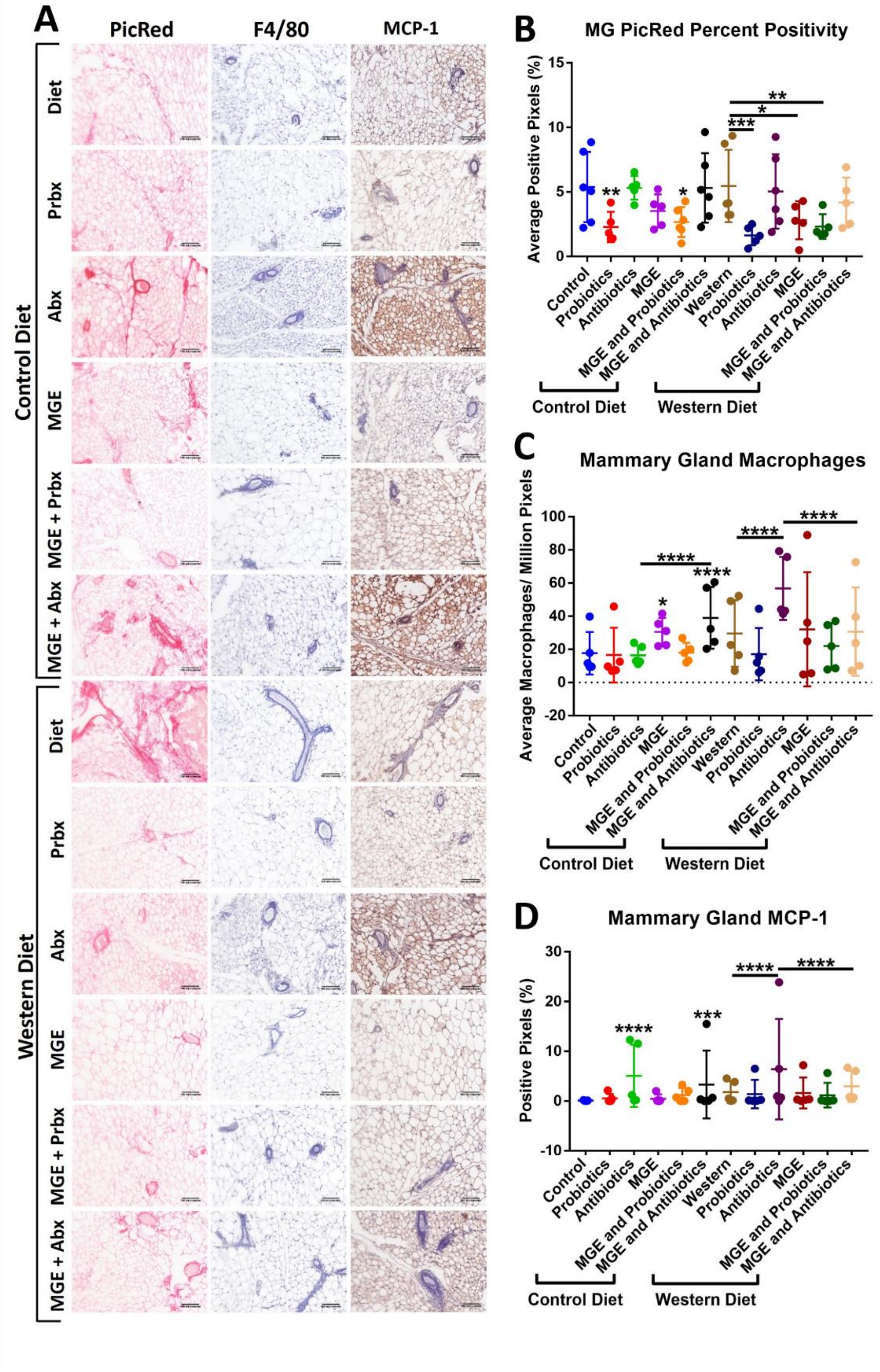

Supplemental Figure S6. Average intestinal muscularis thickness calculated from measurements of the H&E images.

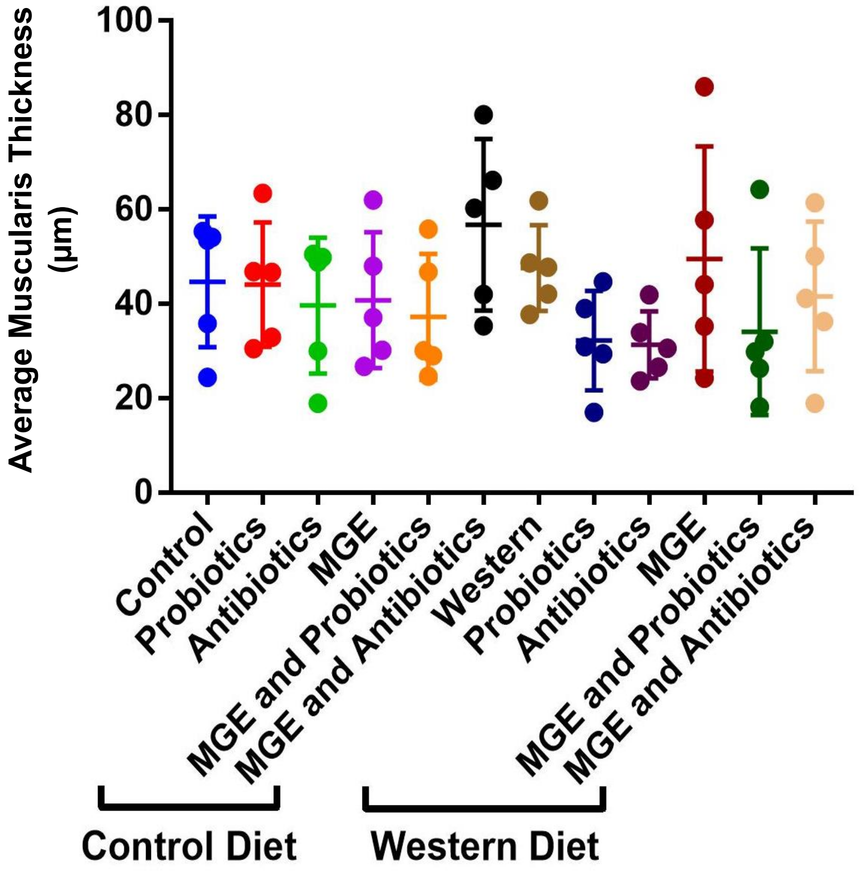

Supplement: Supplementary file 1 [file cells-12-02599-s001.zip › cells-2570099-supplementary.pdf]
